# 2812. Clinical Impact of the Ceftolozane-Tazobactam Shortage on Difficult-To-Treat Resistant *Pseudomonas aeruginosa* Infections at an Academic Medical Center

**DOI:** 10.1093/ofid/ofad500.2423

**Published:** 2023-11-27

**Authors:** Mary K Vance, Christo Cimino, Benjamin Ereshefsky, Romney Humphries, George E Nelson

**Affiliations:** Arkansas Children's Hospital, Little Rock, Arkansas; Vanderbilt University Medical Center, Nashville, Tennessee; Vanderbilt University Medical Center, Nashville, Tennessee; Vanderbilt University Medical Center, Nashville, Tennessee; Vanderbilt University Medical Center, Nashville, Tennessee

## Abstract

**Background:**

Difficult-to-treat resistant *Pseudomonas aeruginosa* (DTR PA) infections are a serious threat, requiring treatment with ceftolozane-tazobactam (C/T), ceftazidime-avibactam, or imipenem-cilastatin-relebactam due to extensive resistance. C/T was unavailable January to December 2021 so alternative agents were used. This study compared clinical outcomes in patients with documented DTR PA infections during periods of availability and shortage.

**Methods:**

This retrospective cohort study included adult patients admitted to Vanderbilt University Medical Center January 2019 to December 2022 who were treated for DTR PA. Patients admitted January to December 2021 were included in the C/T shortage group and all others were included in the C/T available group. DTR PA was defined as nonsusceptibility to fluoroquinolones and traditional antipseudomonal β-lactams. Patients who received antibiotics < 48 hours or were transferred to an outside facility or hospice prior to completing therapy were excluded. The primary outcome was a composite of 90-day survival and clinical success, defined as improvement or resolution of signs of infection such that no further treatment was needed. Secondary outcomes included infection-related mortality, emergence of resistance, and adverse events.

**Results:**

A total of 29 patients were included, 22 in the C/T available group and 7 in the C/T shortage group. DTR PA was most commonly isolated from respiratory (31%) and wound cultures (31%). Significantly more patients in the C/T shortage group received combination therapy (57% vs. 9%, p=0.018). The primary outcome occurred in 50% of those in the C/T available group versus 71% of those in the C/T shortage group, which was not a significant difference (p=0.41). There were also no significant between group differences in any of the secondary outcomes, including infection-related mortality (14% vs. 14%, p=1), resistance development (5% vs. 0%, p=1), and adverse events (18% vs. 14%, p=1).

**Figure:**
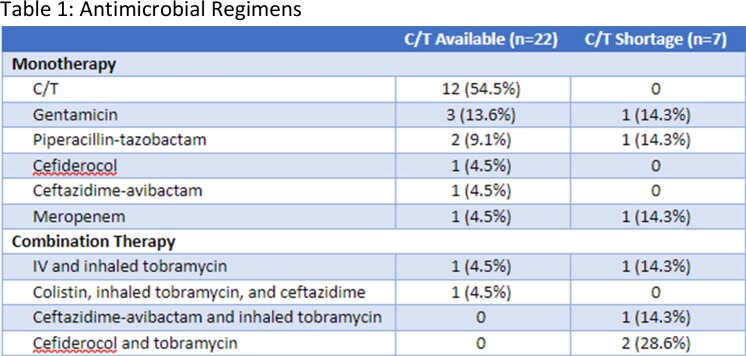

**Conclusion:**

No significant differences were seen in clinical outcomes between patients with DTR PA treated during C/T shortage and available periods. This analysis was limited by a small sample size. Further studies are needed to assess the efficacy of C/T compared to alternative therapy and the role of combination therapy for DTR PA.

**Disclosures:**

**Romney Humphries, PhD, D(ABMM), M(ASCP)**, Melinta: Advisor/Consultant|Merck: Advisor/Consultant|Shionogi: Advisor/Consultant|Ventorx: Advisor/Consultant

